# Effects of Capsaicin on the Growth, Development, and Nutritional Profiles of Black Soldier Flies Fed a High-Fat Diet

**DOI:** 10.3390/insects17060567

**Published:** 2026-05-29

**Authors:** Hao-Rui Gao, Jing-Ze Yuan, Yao Xiao, Jiang-Bo Zhang

**Affiliations:** 1 College of Animal Science and Technology, Northwest A&F University, Yangling 712100, China; 13947274552@163.com; 2 College of Life Science, Northwest A&F University, Yangling 712100, China; 3 State Key Laboratory of Crop Stress Biology for Arid Areas, Northwest A&F University, Yangling 712100, China; yuanjz6677@foxmail.com (J.-Z.Y.); dunwang@foxmail.com (Y.X.)

**Keywords:** black soldier fly, capsaicin, high-fat diet, resource utilization of food waste

## Abstract

This study evaluated the effects of capsaicin on the growth and development and the nutritional composition of black soldier fly larvae (BSFL) under high-fat substrate conditions. The results showed that, under a high-fat background, low levels of capsaicin (0.01–0.05%) had a relatively small adverse effect on the growth of larvae, while 0.1–0.2% treatment led to a decrease in the accumulation of larval weight. The pupation age did not show a monotonous change consistent with the addition amount of capsaicin. The conversion efficiency of digested feed in the high-fat control group was lower than that in the basal diet control group. It slightly increased with 0.01–0.05% capsaicin treatment and decreased again with 0.2% treatment. The amino acid composition of the insect body is relatively stable overall, but the contents of glutamic acid, tyrosine, etc., decrease under high doses of capsaicin. The changes in fatty acid composition are more sensitive, mainly manifested as fluctuations in the ratios of C12:0, C18:1n-9, and C18:2n-6. The results indicate that black soldier flies have a certain tolerance to spicy and high-fat substrates, but when the addition of capsaicin increases, their growth and nutrient deposition will be adversely affected.

## Simple Summary

Nowadays, the harmless treatment and resource utilization of food waste have become important issues for cities. Black soldier flies can efficiently utilize food waste and convert it into insect biomass rich in protein and lipids. Chinese food waste contains a large amount of high-fat and highly spicy components, but research on the comprehensive effects of capsaicin on the growth and development, material transformation efficiency, and nutritional composition of black soldier fly larvae under a high-fat background is still relatively scarce. The research results show that under high-fat feed conditions, low levels of capsaicin (0.01% to 0.05%) have relatively small effects on the growth, development, and transformation efficiency of black soldier fly larvae, while 0.1% to 0.2% treatment weakens the promoting effect of high-fat substrates on the growth of larvae. High-dose capsaicin can reduce the proportion of major amino acids in some parts of the insect body, and the fatty acid composition of the insect body is also sensitive to changes in capsaicin, mainly manifested as fluctuations in the ratios of C12:0, C18:1n-9, and C18:2n-6. The results can provide a reference for the substrate regulation of the black soldier fly in the treatment of spicy and high-fat food waste.

## 1. Introduction

With the acceleration of urbanization and the continuous expansion of population size, the treatment of organic waste such as food waste has become one of the severe challenges faced by the sustainable development of Chinese cities. According to statistics, the annual production of food waste in China has exceeded 63 million tons, and it is expected to reach 73 million tons during the 14th Five-Year Plan period, showing a relatively fast growth trend, and the daily output of food waste in metropolises is huge [1]. If calculated based on 0.3 to 0.5 kg of food waste per person per day, metropolises with a permanent resident population of 5 to 10 million can generate 1500 to 5000 tons of food waste per day, but their processing capacity is only 100 to 300 tons, and the effective rate of resource utilization is even less than 10%. At present, the main technologies for treating food waste include landfills, composting, and anaerobic digestion, etc. However, they have disadvantages such as large land occupation, high pollution risk, and high environmental requirements [2]. Meanwhile, insect breeding, as an emerging method for handling food waste, has undergone extensive experiments and research. As one of the biotransformation technologies for farmed insects, the method of treating food waste by raising BSFL has gradually been adopted by some metropolises in China [3,4,5,6].

The black soldier fly (*Hermetia illucens* L.) belongs to the family Stratiomyidae in the order Diptera. As a holometabolous insect, its life cycle consists of four stages: egg, larva, pupa, and adult [7]. Black soldier flies have a wide range of organic waste recipes, and they can bioconvert food waste and various livestock and poultry manure to obtain high-value insect protein (with dry insect protein content reaching up to 45%), which can be used as a new protein source to replace imported soy protein and fish meal protein [8,9,10]. In addition, black soldier flies have a high conversion efficiency, with a dry matter conversion rate of up to 40% to 60%, and they have broad prospects in both environmental protection and agricultural production fields [11].

Capsaicin is a typical factor in the Chinese diet, widely present in kitchen waste, and its concentration varies significantly due to regional differences in dietary habits. Studies [12,13,14,15,16] have shown that capsaicin in feed can affect indicators such as the pupation rate, emergence rate, and survival rate of BSFL. However, systematic research on its effects on the growth performance, transformation efficiency, and nutritional components of BSFL is still insufficient. A high-fat diet is usually rich in a large amount of saturated fatty acids, and excessive intake may lead to metabolic disorders. Studies have shown that feeding fruit flies a high-fat diet leads to obesity, delayed development, reduced motor ability, increased oxidative stress, a shortened lifespan, and decreased reproductive capacity in both larvae and adults. However, research on the effects of a high-fat diet on the growth and development, nutrient accumulation, and metabolism of the black soldier fly is still very scarce [17,18,19,20].

At present, although some studies have focused on the impact of the characteristics of food waste (such as spiciness) on BSFL [13], and some have revealed the negative effects of a high-fat diet on model insects like fruit flies [12], there has been no study that combines the two. Therefore, this study focuses on the combined effect of capsaicin and feed fat levels on the growth performance and conventional nutrients of BSFL, with the aim of providing a scientific basis and technical reference for the efficient conversion of specific food waste rich in oil and pungent components.

## 2. Materials and Methods

### 2.1. Breeding Method of BSFL

The experiment involved approximately 3600 BSFL. The standard formula (control group) was formulated with 40% soybean meal and 60% wheat bran as the basic feed. The high-fat formula was formulated by adding 10% oil to the standard feed, consisting of 36.3% soybean meal, 54.5% wheat bran, and 9.09% soybean oil (Table 1).

The main instruments and equipment included: electronic balance (ME204E, Mettler Toledo, Greifensee, Switzerland), high-speed crusher (FW100, Tianjin Test Instrument Co., Ltd., Tianjin, China), electric heating constant-temperature air-drying oven (DHG-9140A, Shanghai Yiheng Scientific Instrument Co., Ltd., Shanghai, China), Kjeltec nitrogen determinator (Kjeltec 8400, FOSS, Hillerød, Denmark), Soxhlet extractor (SOX406, Haineng Future Technology Group Co., Ltd., Jinan, China), gas chromatograph (7890B, Agilent, Santa Clara, CA, USA, with flame ionization detector), and amino acid analyzer (L-8900, Hitachi, Tokyo, Japan). All instruments were calibrated or had their performance checked in accordance with the routine requirements before use.

According to the capsaicin concentration in Sichuan cuisine in the literature [17], the capsaicin concentration in extremely spicy dishes is 0.4023 g/kg. Combined with the capsaicin content in dried chili peppers and their products [11], the total capsaicin content in *Capsicum annuum* L. is 6.58 g/kg. Based on this, in this study, six experimental groups were designed, with capsaicin concentrations of 0 (standard diet, CK), 0 (high-fat diet, HF-0), 0.01%, 0.05%, 0.1%, and 0.2% (all high-fat diets, HF-0.01, HF-0.05, HF-0.1, and HF-0.2), respectively. Each experimental group was set with 3 replicates, totaling 18 groups. After the high-fat diet was prepared, the mass concentration of capsaicin was adjusted using capsaicin, and the prepared diet was sealed and set aside for later use.

The larvae were respectively introduced into food cans (polypropylene, 15 cm × 10 cm × 5 cm) containing six different concentrations of capsaicin. Each food can was regarded as one repeat, and the number of larvae raised was 200. The numbered cans were placed on the breeding rack; the breeding environment temperature was controlled at 28 ± 2 °C and the relative humidity at about 80%. The growth of the larvae was observed regularly every day and replenished with a certain amount of distilled water to maintain the humidity. The initial weight of the bait in each jar was 50 g, with a moisture content of 70%. The consumption of the bait was confirmed and replenished in time. The total weight of the diet added to each group was controlled to be consistent.

### 2.2. Determination of Relevant Indicators

The larval period was the number of days (d) taken when half of the pre-pupa appears. The first day was recorded when the larvae and feed were placed together in the culture cans. The feeding experiment ended when 50% of the larvae entered the pre-pupa state, and the number of days of the experiment was recorded.

At the beginning of the experiment, the total wet weight of the larvae was recorded. Then, every 2 days, 10 larvae were randomly selected for each repetition. After weighing the wet weight, they were put back into the food cans for continued rearing. At the end of the experiment, the larvae were carefully screened out by hand with sterile tweezers to measure their final wet weight, and the average weight gain rate, growth rate, and total weight gain of the larvae were obtained. The average weight gain rate was the ratio of the wet weight of the larvae to the base number of 100, and the growth rate was the ratio of the wet weight of the larvae to the number of days of rearing. At the end of the experiment, the number of surviving larvae was recorded. With 100 larvae as the ideal survival value, the larval survival rate was calculated.

After the rearing was completed, the larvae and the remaining substances separated from the larvae were dried at 105 °C and then weighed. The conversion (efficiency of conversion of digested food ECD) was the ratio of the total dry weight increase in BSFL to the total dry weight decrease in feed and was calculated using the following equation [21]:

ECD = m4/(m2 − m5)(1)

where m4 was the total dry weight (g) increase in the larvae at the end of the experiment, m2 was the total dry weight (g) of the feed added during the experiment, and m5 was the total dry weight (g) of the remaining substrate at the end of the test.

After BSFL samples were heated and dried at 105 °C, the weight lost was the moisture content, and the remaining weight was the amount of dry matter according to the dry matter calculation equation [11]. To reduce the interference of intestinal contents, the larvae were starved for 12 h after the experiment. The surface attachments were rinsed with distilled water, and the larvae were weighed after drying on filter paper. The dried larval samples were crushed, mixed evenly, and then placed in sealed bags for subsequent determination of amino acids and fatty acids.

Crude protein was determined using the Kjeldahl nitrogen determination method, with the nitrogen content multiplied by 6.25 for calculation [22]. The determination of amino acids was carried out in accordance with the conventional feed analysis method: after the samples were hydrolyzed with 6 mol/L hydrochloric acid at 110 °C for 24 h, the content of each amino acid was determined by an amino acid analyzer, and the results were expressed on a dry matter basis. Tryptophan was easily destroyed by acid hydrolysis, so it was not reported separately in this study.

Crude fat was determined by Soxhlet extraction [23]. When determining the composition of fatty acids, lipid samples were taken and subjected to methylation treatment. The peak areas of each fatty acid methyl ester were analyzed by gas chromatography, and quantification was carried out by internal standard method or area normalization method. The chromatographic column adopted was an HP-88 capillary column (100 m × 0.25 mm × 0.20 μm), and the detector was a flame ionization detector. The results were expressed as the percentage of each fatty acid in the total fatty acids. Each sample was determined in parallel at least twice.

### 2.3. Statistical Analysis

The obtained data were processed using Excel 2019 and IBM SPSS Statistics 26, and the data presentation was in the form of mean ± standard deviation. The normality of each parameter was tested using the Shapiro–Wilk test (*p* < 0.05). Data conforming to the normal distribution were analyzed using one-way analysis of variance (*p* < 0.05). In this case, if equal variances were assumed, the Tukey test was used; if no equal variances were assumed, the Dunnett’s test was used. For data with non-normal distribution, the Mann–Whitney U test was used (*p* < 0.05).

## 3. Results

### 3.1. The Growth and Development of BSFL

**Figure 1:**
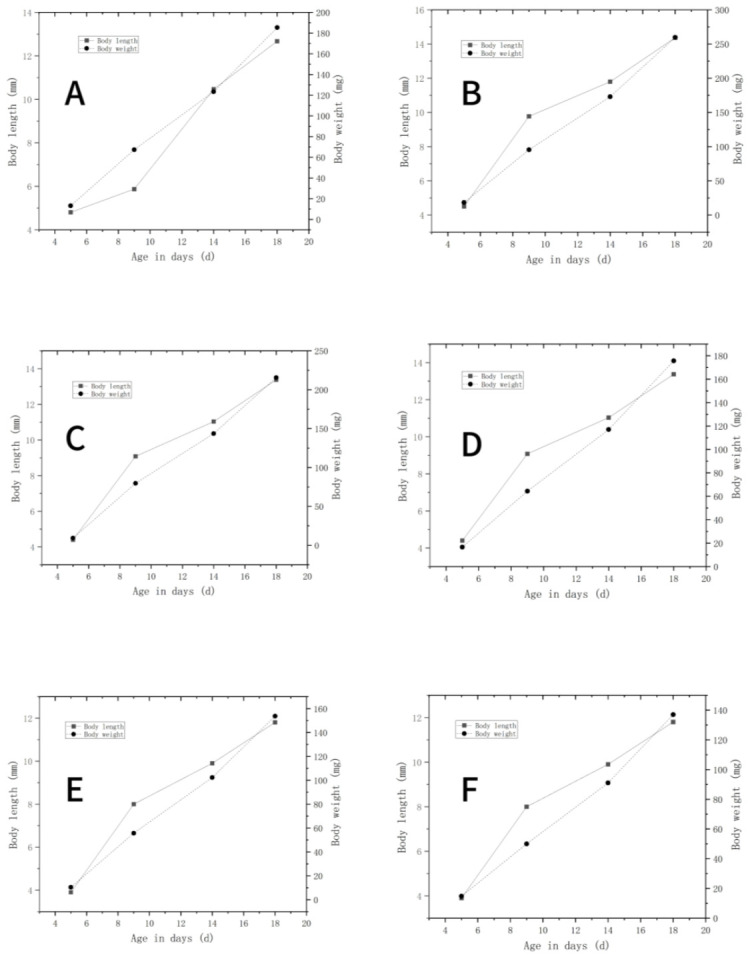
Line chart of growth rate of black soldier fly (*Hermetia illucens* L.) larvae in different experimental groups. (**A**) Group CK; (**B**) Group HF-0; (**C**) Group HF-0.01; (**D**) Group HF-0.05; (**E**) Group HF-0.1; and (**F**) Group HF-0.2. CK: control group; HF: high-fat groups.

Figure 1 and Table 2 show the changes in the body length, weight, and related growth indicators of BSFL in different treatment groups. Overall, the body length and weight of the larvae in each treatment group increased with the increase in age, but there were significant differences in the growth rates among different treatments. The overall growth performance of the high-fat control group (HF-0) was better than that of the basal diet control group (CK). After the addition of capsaicin under a high-fat background, the growth dominance of the larvae gradually weakened.

Generally, compared with the CK group, the total weight gain was significantly increased or decreased in the HF-0, HF-0.01, HF-0.1, and HF-0.2 groups (*p* < 0.05), among which there was no significant difference in the HF-0.05 group. The quantity only increased significantly in the HF-0.01 group (*p* < 0.05). The average weight gain was extremely significantly increased in the HF-0 group (*p* < 0.001), significantly decreased in the HF-0.1 group (*p* < 0.05), and significantly decreased in the HF-0.2 group (*p* < 0.01). The weight gain rate increased extremely significantly in the HF-0 group (*p* < 0.001), increased significantly in the HF-0.01 and HF-0.1 groups (*p* < 0.05), and decreased extremely significantly in the HF-0.2 group (*p* < 0.001). The average growth was only significantly higher in the HF-0 group than in the CK group (*p* < 0.01). There was no significant difference in pupation age between each group and the CK.

As can be seen from Figure 1, the body length and weight of the larvae in the CK group increased steadily with age. The HF-0 group showed a relatively fast body weight accumulation in each measurement period, and the body weight of the larvae at the last stage was the highest, indicating that moderately increasing the fat level is beneficial to the formation of insect biomass. The growth curves of the HF-0.01 group and the HF-0.05 group were generally similar to those of the HF-0 group, but the body weight of the late-stage larvae was slightly lower. When the addition amount of capsaicin was increased to 0.1% and 0.2%, respectively, both the body length and weight growth of the larvae slowed down significantly. Although the HF-0.1 group showed a trend of entering the pre-pupa stage earlier, the weight accumulation of the late-stage larvae was insufficient. The growth rate of the HF-0.2 group was relatively low throughout the observation period, indicating that a higher capsaicin level had a sustained inhibitory effect on the growth of BSFL under high-fat conditions.

As can be seen from Table 2, the pupation age in each area is concentrated between 19 and 21 days, and the differences among the groups are generally not significant. The age of pupae in the CK group was 20.0 days, while that in the HF-0 group was prolonged to 21.0 days. After the addition of capsaicin, the HF-0.01 and HF-0.05 groups basically returned to around 20 days, the HF-0.1 group shortened to 19.0 days, and the HF-0.2 group rebounded to 20.3 days. It is indicated that, under the conditions of this experiment, the effect of capsaicin on the developmental rhythm of larvae under high-fat substrates did not show a stable linear change. In terms of the survival rate, the survival rates of each treatment ranged from 93.5% to 99.0%. Among them, the survival rate of the HF-0.01 group was the highest and that of the HF-0.2 group was the lowest. However, the overall fluctuation range was limited, indicating that the larvae of black soldier flies still had a certain tolerance to capsaicin within the set range of this experiment.

The average weight gain per worm in the HF-0 group was the highest at 259.7 mg, which was significantly higher than 185.3 mg in the CK group. With the increase in the capsaicin addition amount, the average weight gain per insect generally showed a downward trend, and it decreased to 137.0 mg in the HF-0.2 group. The change in the average increment of body length was basically consistent with this, with the largest in the HF-0 group and the lower ones in the HF-0.1 and HF-0.2 groups. The results show that, under a high-fat background, low levels of capsaicin have a relatively small impact on growth volume, while medium to high levels of capsaicin weaken the growth-promoting effect brought by high-fat feed.

### 3.2. Changes in ECD of BSFL

**Figure 2:**
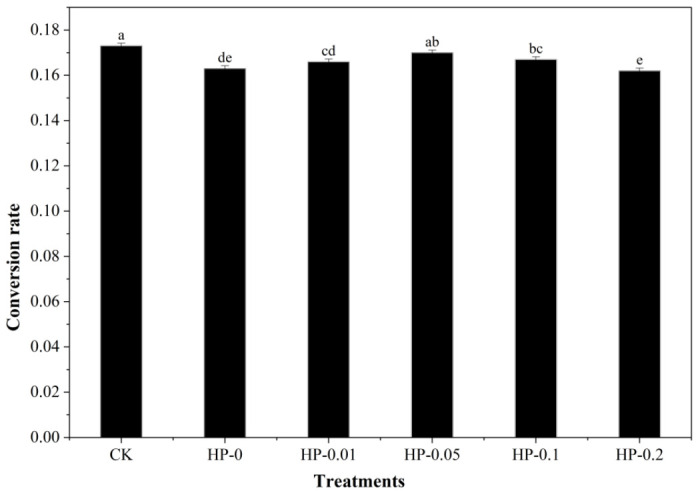
Efficiency of conversion of digested feed (ECD) of BSFL under different dietary treatments: basal diet control (CK), high-fat control (HF-0), and high-fat diet supplemented with 0.01%, 0.05%, 0.1%, or 0.2% capsaicin (HF-0.01 to HF-0.2). The error line represents the standard deviation (SD). Different letters indicated significant differences between treatment groups (*p* < 0.05), and one-way analysis of variance (ANOVA) was used, followed by Tukey HSD test (CK: 0.175, HF-0: 0.164, HF-0.01: 0.166, HF-0.05: 0.168, HF-0.1: 0.167, and HF-0.2: 0.165).

The conversion ability of substrates to insect biomass in different treatment groups was evaluated by ECD, and the results are shown in Figure 2. The ECD in the CK group was 0.175 and that in the HF-0 group decreased to 0.164, indicating that simply increasing the fat level did not simultaneously enhance the conversion efficiency of substrates to insect biomass.

One-way analysis of variance indicated that there were extremely significant differences in conversion rates among different treatment groups (*p* < 0.001). Compared with the CK group, the conversion rates of all high-fat treatment groups were significantly reduced: among them, the reduction in the HF-0, HF-0.01, HF-0.1, and HF-0.2 groups reached an extremely significant level (*p* < 0.001), and the reduction in the HF-0.05 group reached a significant level (*p* < 0.05). Within each HF treatment group, the conversion rate of the HF-0.05 group was significantly higher than that of the HF-0 group (*p* < 0.01), the HF-0.01 group (*p* < 0.05), and the HF-0.2 group (*p* < 0.01), but there was no significant difference compared with the HF-0.1 group (*p* > 0.05). The conversion rate of the HF-0.1 group was significantly higher than that of the HF-0.2 group (*p* < 0.05). There were no significant differences between the HF-0 group and the HF-0.2 group or between the HF-0.01 group and the HF-0.1 group (*p* > 0.05). The above results indicate that a simple high-fat feed (HF-0) can reduce the conversion rate, while adding an appropriate amount of capsaicin (0.05%) can partially alleviate this downward trend. However, a high concentration (0.2%) of capsaicin further inhibits the conversion efficiency, suggesting that the effect of capsaicin on the conversion rate is non-monotonic and concentration-dependent.

After capsaicin was added to the high-fat feed, the ECD in the HF-0.01 group and the HF-0.05 group rebounded to 0.166 and 0.171, respectively. The ECD in the HF-0.1 group was close to that in the HF-0.05 group, while that in the HF-0.2 group dropped again to approximately 0.160. Overall, low levels of capsaicin have a relatively small impact on the conversion efficiency under high-fat conditions, and there is even a certain upward trend. However, when the addition amount increased to 0.2%, the conversion efficiency decreased, suggesting that a higher capsaicin level was not conducive to substrate utilization.

### 3.3. Changes in the Nutritional Components of BSFL

Table 3 shows the amino acid composition of BSFL in different treatment groups. Overall, the amino acid profiles did not undergo fundamental changes among the treatments, but the response intensities of different amino acids to capsaicin varied. Compared with the basal diet control group, the content of some amino acids in the high-fat control group changed little. After further increasing the capsaicin level under the background of high fat, some amino acids showed a downward trend, being especially more obvious in the HF-0.2 group.

Compared with the CK group, Phe was significantly increased in the HF-0.1 and HF-0.2 groups (*p* < 0.05); Thr was significantly decreased in the HF-0.1 group (*p* < 0.05) and extremely significantly decreased in the HF-0.2 group (*p* < 0.01). Ala was significantly decreased in the HF-0.01, HF-0.05, and HF-0.1 groups (*p* < 0.05) and extremely significantly decreased in the HF-0.2 group (*p* < 0.01). Arg was extremely significantly decreased in all capsaicin-containing treatment groups (HF-0.01 to 0.2) (*p* < 0.001); Glu was significantly decreased in the HF-0 group (*p* < 0.05), extremely significantly decreased in the HF-0.01 group (*p* < 0.01), and extremely significantly decreased in the HF-0.05, HF-0.1, and HF-0.2 groups (*p* < 0.001); and Tyr was extremely significantly decreased in the HF-0.01, HF-0.1, and HF-0.2 groups (*p* < 0.001) and extremely significantly decreased in the HF-0.05 group (*p* < 0.01). Cys-Cys significantly increased in the HF-0 group (*p* < 0.05), extremely significantly increased in the HF-0.1 group (*p* < 0.001), and extremely significantly increased in the HF-0.2 group (*p* < 0.01). The remaining amino acids and treatments showed no significant differences from CK (*p* > 0.05).

Among essential amino acids, lysine, methionine, and threonine account for relatively high proportions, while isoleucine, leucine, and valine have relatively low contents. Compared with the CK group, the changes in most essential amino acids in the HF-0 group were limited. With the increase in the capsaicin addition amount, isoleucine, threonine, etc., showed a downward trend, while lysine and methionine remained relatively stable at low to medium addition levels, and they only showed a more obvious decrease in the HF-0.2 group. This indicates that high-dose capsaicin has an adverse effect on the deposition of some essential amino acids.

On the other hand, among non-essential amino acids, glutamic acid and tyrosine have relatively high contents and are the main components of each treatment group. Table 3 shows that glutamic acid and tyrosine decreased significantly in the HF-0.1 and HF-0.2 groups (*p* < 0.001). Proline and arginine also show a certain downward trend. In contrast, glycine, valine, and phenylalanine changed less, indicating that the sensitivity of different amino acids to capsaicin stress is not consistent.

As for other amino acids, cystine showed little change under low-dose treatment but tended to increase in the HF-0.1 and HF-0.2 groups. Histidine slightly decreased under high-dose treatment (*p* > 0.05). The above results suggest that under a high-fat background, the effect of capsaicin on the amino acid composition of insects is mainly manifested as a redistribution of the proportion of some amino acids rather than an overall synchronous decrease.

Table 4 shows the changes in the fatty acid composition of BSFL in different treatment groups. Compared with amino acids, the fatty acid composition is more sensitive to the addition of high-fat substrates and capsaicin. Overall, the main fatty acid components of each treatment were C12:0, C16:0, C18:1n-9, and C18:2n-6, but their relative proportions fluctuated with different treatments.

Compared with the CK group, C12:0 was extremely significantly increased in the HF-0, HF-0.1, and HF-0.2 groups (*p* < 0.001) and significantly decreased in the HF-0.01 group (*p* < 0.05); C14:0 was extremely significantly increased in the HF-0.1 group (*p* < 0.01); C18:0 was significantly decreased in the HF-0.1 group (*p* < 0.05); C18:1n-9 was extremely significantly decreased in the HF-0 group (*p* < 0.01) and extremely significantly decreased in the HF-0.1 and HF-0.2 groups (*p* < 0.001); and C18:2n-6 was significantly decreased in the HF-0 group (*p* < 0.05), extremely significantly decreased in the HF-0.1 group (*p* < 0.001), and extremely significantly decreased in the HF-0.2 group (*p* < 0.01). The remaining fatty acids showed no significant differences from the CK in each treatment group (*p* > 0.05). For fatty acids with a standard deviation of 0 in the CK group (such as C10:0, C13:0, C14:1, C18:3n-6, etc.), even if there are non-zero values in the treatment group, the significance is not marked, as *t*-tests cannot be conducted.

Among saturated fatty acids, the C12:0 change is the most obvious. Compared with the CK group, C12:0 was significantly increased after high-fat treatment and reached the highest value in the HF-0.1 group (*p* < 0.05). Although the HF-0.2 group was lower than the HF-0.1 group, it was still higher than the CK group. C16:0 maintained a relatively high proportion in each treatment group, and the fluctuations between groups were relatively small. C14:0 and C18:0 showed moderate changes. The results indicate that, when high-fat substrates are superimposed with capsaicin, the deposition patterns of medium-chain and long-chain saturated fatty acids in the insect body will be adjusted.

Monounsaturated fatty acids are mainly composed of C18:1n-9. The content of C18:1n-9 was relatively high in the CK, HF-0, and HF-0.05 groups, while it decreased significantly in the HF-0.1 and HF-0.2 groups (*p* < 0.001), suggesting that a higher level of capsaicin may inhibit the accumulation of this fatty acid. C16:1 also fluctuated among various treatments, but the amplitude was smaller than that of C18:1n-9.

Among polyunsaturated fatty acids, C18:2n-6 is the main component. Under low to moderate capsaicin treatment, C18:2n-6 remained at a relatively high level; when capsaicin rose to 0.1% and 0.2%, its proportion decreased significantly (*p* < 0.01). The content of C20:4n-6 was relatively low in each treatment but slightly increased in the HF-0.01 group (*p* > 0.05). Overall, this indicates that high-dose capsaicin not only inhibits the growth of larvae but also alters the deposition characteristics of unsaturated fatty acids.

## 4. Discussion

This study explored the effects of different concentrations of capsaicin on the growth and development, survival rate, transformation efficiency, and nutritional components of BSFL under the background of a high-fat diet. The results indicated that the addition of capsaicin inhibited the growth performance of larvae to a certain extent, and the high-concentration group (0.2%) showed significant growth retardation and decreased transformation efficiency, suggesting that capsaicin may act as a stress factor to affect the physiological metabolism of black soldier flies. Ji reported that BSFL have an overall good tolerance to pH, salinity, and spiciness in food, indicating that they have the basic biological potential to handle complex food waste [15]. Yang further found that, under the condition of simple spiciness treatment, there were no significant differences in most growth performance, survival rate, and waste reduction indicators. However, the conversion efficiency of the 0.6% capsaicin group was the lowest, and the 0.8% capsaicin group had the least conversion of feed into nutrients for the insects [24]. Compared with the above results, in this study, under a high-fat background, the effect of capsaicin on BSFL shows obvious dose characteristics. Considering the pupation age and survival status, the main impact of capsaicin is not to cause large-scale deaths but is more likely to be manifested as a decline in feeding and nutrient deposition efficiency, thereby weakening the formation of larval biomass.

A moderate amount of fat can provide energy sources for larvae and promote their growth and development, but excessive fat may cause metabolic disorders. In the study of feeding fruit flies with high-fat feed, it was found that excessive fat intake can lead to the delayed development of larvae and increased oxidative stress [14]. In this study, the body weight of the larvae in the high-fat control group (0% capsaicin) slightly increased, but the conversion rate did not significantly improve, indicating that simply increasing the fat content cannot effectively enhance the biotransformation efficiency of black soldier flies. Zhang et al. [25] found that the fat content in the substrate has a dual effect on the larvae of black soldier flies: an appropriate amount of fat can promote the growth of larvae and the accumulation of biomass, but excessive fat or combination with other adverse factors (such as the deterioration of the physical and chemical properties of the substrate) will instead inhibit the transformation efficiency. These results are highly consistent with ours. We also observed, in a high-fat background (with 10% soybean oil added), that although simply increasing the fat level could increase the weight of the larvae, it did not simultaneously enhance the transformation efficiency (ECD decreased from 0.175 to 0.164). However, after further addition of capsaicin (0.1–0.2%), the growth-promoting effect of high fat was significantly weakened, the transformation efficiency further declined, and the fatty acid composition (the ratio of C12:0, C18:1n-9, and C18:2n-6) also fluctuated significantly.

Capsaicin has an inhibitory effect on the pupation rate and emergence rate of BSFL but has a relatively small impact on the growth performance during the larval stage [15]. However, this study found that, after adding capsaicin to a high-fat diet, both the weight gain and survival rate of larvae were significantly inhibited, especially at concentrations of 0.1% and 0.2%, where the larval period was prolonged and the conversion rate decreased. Studies have shown that environmental factors such as pH have a phased impact on the growth of larvae, and capsaicin may produce a similar effect by altering the intestinal microenvironment. High-concentration capsaicin treatment prolonged the time for larvae to reach the pre-pupa stage, indicating that capsaicin may interfere with the normal growth sequence of black soldier flies. Previous studies have shown that changes in the physical and chemical properties of the substrate can significantly affect the developmental process of black soldier flies [15,26,27]. Compared with the black soldier fly observed by Sanjaya et al. that completed its life cycle in 29–41 days under suitable conditions [26], the developmental process of the capsaicin treatment group in this experiment was significantly delayed, suggesting that capsaicin may have interfered with the normal developmental sequence of the larvae. Zhang Jiabao et al. pointed out that the growth and development of larvae are better under conditions of pH6–10 and a salt concentration within 6% [27].

It is worth noting that the age of pupation did not show a single, monotonous change with the addition level of capsaicin (Table 2). This dual-phase effect of “low-dose stimulation and high-dose inhibition” is precisely a typical feature of the excitatory effect of toxins. The systematic review by Rix and Cutler [28] indicates that such biphase responses are widespread in insects and can manifest as phenotypes such as accelerated growth and development, an enhanced reproductive capacity, prolonged lifespan, and improved tolerance to environmental stress. This review further reveals that the molecular pathways related to the excitatory effect of toxins mainly include alterations in antioxidant enzyme activity, the upregulation of heat shock proteins, enhanced expression of detoxification genes, and changes in gene expression in the IIS/TOR signaling pathway that directly regulates growth, development, and reproduction. In addition, the changes in the levels of juvenile hormones and vitellogenin induced by stress are also associated with the toxic excitatory effect. Cutler et al. also pointed out that mild chemical, high temperature, or nutritional stress can all induce excitatory effects in insects [28,29]. In addition to hormone regulatory pathways, energy redistribution is also an important dimension for understanding the mechanism of the excitatory effect of poisons. In this study, the weight gain rate of the high-dose capsaicin treatment group decreased significantly (*p* < 0.001) (the final weight of the HF-0.2 group was only 23% of that of the HF-0 group), suggesting that capsaicin stress may interfere with the feeding or digestive absorption efficiency of larvae. Under the state of “perceived nutritional deficiency”, according to the discussion by Rix and Cutler on the core role of the IIS/TOR signaling pathway in the toxic excitation effect, larvae may, by downregulating the activity of this pathway, prioritize the allocation of limited energy reserves to stress defense responses and homeostasis maintenance rather than to developmental processes and tissue growth [28]. Based on the above analysis, we believed that the possible reason for the non-monotonic developmental response observed in this study was that 0.1% capsaicin, as a mild source of chemical stress, may indirectly promote the synthesis and release of the molting hormone by moderately activating the antioxidant defense system and IIS/TOR signaling pathway of the larvae, thereby enabling the larvae to enter the pre-pupa stage earlier. When the capsaicin dose rose to 0.2%, the oxidative stress and metabolic burden caused by excessive stress exceeded the compensatory capacity of the larvae, resulting in the inhibition of developmental initiation and the recovery of the pupation age to the level of the high-fat control group.

From the perspective of nutritional components, the addition of capsaicin did not significantly change the crude protein and crude fat content of the insects, but the fatty acid composition of the high-concentration group changed to some extent. The ratio of C12:0 (lauric acid) slightly decreased, while the ratios of C18:2 and C18:1 increased. Liu Yanxia’s research indicates that the substrate composition significantly affects the fatty acid composition in black soldier flies. The larvae preferentially convert carbohydrates into their own fatty acids and only start to utilize waste oils when the carbohydrates are nearly exhausted [13]. In this study, the high-fat diet itself may have altered the lipid metabolism pathway of the larvae, and the addition of capsaicin further exacerbated this metabolic disturbance. Under the combined action of high-fat substrates and capsaicin, the first thing that may occur in BSFL is the adjustment of lipid deposition pathways and fatty acid distribution patterns rather than the overall remodeling of the protein composition. It should be noted that the crude protein and crude fat content of the larvae were not determined in this study, which limits the comprehensive evaluation of the nutritional quality of the larvae. Subsequent studies should combine the determination of crude protein and crude fat with the analysis of amino acids and fatty acids to gain a deeper understanding of the comprehensive impact of capsaicin on the macronutrient composition of black soldier fly larvae. In addition, the residual level of capsaicin in the larvae was not determined in this study. Subsequently, methods such as LC-MS/MS need to be adopted to further explore its metabolic accumulation pattern.

It is also worth noting that the relationship between the addition level of capsaicin and the fatty acid composition of the insect body shows a significant concentration dependence. Tian et al. reported that CYP6B6 and CYP9A series P450 enzymes in the cotton bollworm (*Helicoverpa armigera*) can efficiently convert capsaicin, and hydroxylation and dehydrogenation are its two main metabolic pathways [30]. This discovery provides important clues for understanding the lipid metabolism changes in black soldier flies induced by capsaicin: larvae consume a large amount of energy substrates such as NADPH during the metabolism of capsaicin, and at the same time, the P450 reaction is accompanied by the production of reactive oxygen species, increasing the overall metabolic burden of the larvae. Rix and Cutler also pointed out, in their review of the excitatory effects of poisons, that the enhanced expression of detoxification enzyme genes is one of the molecular bases for insects to respond to mild stress [28]. From the perspective of fatty acid synthesis, Giannetto et al. first systematically characterized the expression of key lipid metabolism genes such as acetyl-coA carboxylase (*acc*), fatty acid synthase (*fas*), lipase (*lip*), and acyl-coA dehydrogenase (*acd*) in black soldier flies [31]. It was also found that the differences in fatty acid profiles at different developmental stages were closely related to the expression regulation of these genes. Capsaicin stress may weaken the synthetic efficiency of C12:0, C18:1n-9, and C18:2n-6 by interfering with the activity of transcription factors such as PPAR/SREBP-1c upstream and inhibiting the expression of key synthetase genes such as *acc* and *fas*. In addition, capsaicin may also directly affect the desaturation process of fatty acids. C18:2n-6, as an essential fatty acid for insects, is highly dependent on Δ12 desaturase for endogenous synthesis when it cannot obtain a sufficient supply from the substrate. Capsaicin-induced oxidative stress may cause functional damage to this enzyme, resulting in a decrease in C18:2n-6 content with the increase in the capsaicin concentration. C12:0 has a unique endogenous synthetic advantage in black soldier flies. In summary, the concentration-dependent effect of capsaicin on the fatty acid composition of black soldier flies can be attributed to three interrelated pathways: (1) The P450 detoxification metabolic process consumes energy substrates and triggers oxidative stress; (2) oxidative stress inhibits the activities of key lipid synthesis enzymes (ACC, FAS) and desaturase; and (3) when the high concentration of capsaicin exceeds the compensatory capacity of the larvae, the process of fatty acid synthesis and desaturation is systematically inhibited.

In addition, changes in substrate composition not only affect growth indicators but also reshape the transformation efficiency and final product quality of black soldier flies. Naser El Deen Somaya found, after comparing various animal and plant wastes and fecal substrates, that the conversion efficiency and waste reduction index of the fast-food waste group were higher, and the fat content of the obtained larvae was also higher [32,33]. In this study, the trend of a slight increase in body weight but a decrease in conversion efficiency in the high-fat control group also indicates that simply increasing the fat supply does not necessarily lead to higher material utilization efficiency. These studies indicate that black soldier flies have a strong plasticity towards substrate components. Therefore, by optimizing feed formulations and environmental conditions, the negative effects of stress factors such as capsaicin can be effectively alleviated. It is recommended that, in production applications, the content of capsaicin in high-fat substrates should not exceed 0.05% (calculated based on the dry weight of feed). When the addition amount of capsaicin reaches 0.1%, larval weight gain has already shown a significant decrease. When it reaches 0.2%, the growth inhibition effect becomes more significant. Therefore, to achieve the optimal growth and transformation efficiency of BSFL, the capsaicin concentration in the substrate should be controlled at 0.05% or less. If the spiciness of kitchen waste raw materials is too high, it is recommended to reduce the capsaicin level below this threshold by mixing and diluting it with other low-spiciness substrates before conducting breeding.

## 5. Conclusions

Under high-fat feed conditions, low levels of capsaicin (0.01% to 0.05%) have a relatively small effect on the growth, development, and transformation efficiency of BSFL. However, 0.1% to 0.2% treatment weakens the promoting effect of high-fat substrates on larval growth, manifested as a decrease in the average weight gain per insect, body length increase, and transformation efficiency. Among them, the inhibitory effect of the 0.2% treatment is the most obvious. The pupation age in various places was generally concentrated between 19 and 21 days, and the survival rate fluctuated little, indicating that the black soldier fly has a tolerance to capsaicin within a certain range. The overall amino acid composition of the insect body is relatively stable, but high-dose capsaicin can reduce the proportion of some major amino acids. The fatty acid composition is more sensitive to treatment changes. C12:0, C18:1n-9, and C18:2n-6 are the fatty acid components that are more significantly affected. The research results show that, when black soldier flies are used to treat spicy and high-fat food waste, the capsaicin and oil load should be controlled to balance the growth of larvae, substrate utilization efficiency, and product quality.

## Figures and Tables

**Table 1:** Composition and nutritional profile of experimental substrates.

Estimated Chemical Composition ^1^	Content of Standard Formula/%	Content of High-Fat Formula/%
Crude protein (CP)	28.0	21.6
Crude fat (EE)	3.0	11.8
Carbohydrates (CHO)	52.0	48.4
Crude fiber	25.7	23.4
Capsaicin ^2^	0	0, 0.01, 0.05, 0.1, 0.2

^1^ Nutritional values were estimated based on literature data for the raw materials: crude protein of soybean meal 44% and wheat bran 14%; crude fat of soybean meal 2.39%, wheat bran 4.25%, and soybean oil 99.9%; and carbohydrates of soybean meal 35.89% and wheat bran 64.51%. The composition of the basal ingredients (soybean meal, wheat bran, and soybean oil) was identical in all high-fat treatments; only the capsaicin level varied. ^2^ Capsaicin (CAS 404-86-4, purity 97%) was dissolved in a small volume of absolute ethanol, pre-mixed with soybean oil, and then thoroughly blended into the high-fat diet using stepwise dilution to ensure uniform distribution.

**Table 2:** Growth performance of black soldier fly (*Hermetia illucens*) larvae under six dietary treatments including capsaicin (0, 0.01, 0.05, 0.1, and 0.2%) and high-fat conditions across different treatment groups.

Treatment Groups	Total Increment/g	Survival Rate (%)	Average Increment per Larva/mg	Total Daily Increment/(mg/d)	Average Increase in Body Length/mm	Pupation Age/d
CK	34.8 ± 0.5 ^c^	94.0 ± 1.35 ^b^	185.3 ± 17 ^b^	1740 ± 77 ^c^	12.7 ± 1.30 ^b^	20.0 ± 0.3 ^b^
HF-0	49.0 ± 0.7 ^a^	95.5 ± 0.85 ^b^	259.7 ± 13 ^a^	2333 ± 109 ^a^	14.4 ± 0.45 ^a^	21.0 ± 0 ^a^
HF-0.01	43.1 ± 0.6 ^b^	99.0 ± 0.65 ^a^	215.7 ± 41 ^ab^	2155 ± 98 ^b^	13.4 ± 1.47 ^ab^	20.0 ± 1.0 ^b^
HF-0.05	34.5 ± 1.0 ^c^	96.0 ± 1.65 ^ab^	175.7 ± 53 ^b^	1725 ± 157 ^c^	12.1 ± 2.48 ^b^	20.0 ± 0.6 ^b^
HF-0.1	30.1 ± 0.06 ^d^	98.0 ± 1.0 ^ab^	153.7 ± 32 ^c^	1584 ± 98 ^d^	11.8 ± 1.44 ^b^	19.0 ± 0.6 ^c^
HF-0.2	25.5 ± 0.2 ^e^	93.5 ± 1.15 ^b^	137.0 ± 8.3 ^c^	1256 ± 33 ^e^	11.5 ± 0.23 ^b^	20.3 ± 1.1 ^b^

Data are expressed as mean ± standard deviation (3 replicates for each treatment). The initial inoculation number for each treatment was 200 individuals per replicate; the survival rate can be calculated from the number of survivors. CK: control group; HF: high-fat groups. One-way analysis of variance (ANOVA) was used to conduct a comprehensive test for each column of indicators. Subsequently, the Tukey HSD test was used to conduct pairwise comparisons among all treatment groups (CK, HF-0, HF-0.01, HF-0.05, HF-0.1, and HF-0.2). In the same column, different superscript letters indicate significant differences between treatment groups (*p* < 0.05), while the same letter indicates no significant differences (*p* > 0.05).

**Table 3:** Amino acid composition (g/100 g dry matter) of BSFL under six dietary treatments: basal diet control (CK), high-fat control (HF-0), and high-fat diet supplemented with 0.01%, 0.05%, 0.1%, or 0.2% capsaicin (HF-0.01 to HF-0.2).

Composition	CK	HF-0	HF-0.01	HF-0.05	HF-0.1	HF-0.2
His	2.5 ± 0.3 ^ab^	2.5 ± 0.3 ^ab^	2.3 ± 0.1 ^ab^	2.3 ± 0 ^b^	2.3 ± 0.1 ^ab^	2.2 ± 0 ^b^
Ile	1.2 ± 0.1 ^bc^	1.3 ± 0.1 ^ab^	1.1 ± 0.1 ^c^	1.2 ± 0.1 ^bc^	1.4 ± 0.1 ^a^	1.4 ± 0.1 ^a^
Leu	1.7 ± 0.1 ^ab^	1.8 ± 0.1 ^a^	1.6 ± 0.1 ^b^	1.6 ± 0.1 ^b^	1.7 ± 0.1 ^ab^	1.6 ± 0.1 ^b^
Lys	3.1 ± 0.2 ^ab^	3.2 ± 0.2 ^ab^	3.0 ± 0.1 ^b^	2.9 ± 0.2 ^b^	3.2 ± 0.1 ^a^	3.1 ± 0.1 ^ab^
Met	3.1 ± 0.2 ^ab^	3.2 ± 0.2 ^ab^	3.0 ± 0.1 ^b^	3.0 ± 0.1 ^b^	3.1 ± 0.1 ^ab^	3.0 ± 0.1 ^b^
Phe	0.4 ± 0.1 ^c^	0.5 ± 0.1 ^bc^	0.5 ± 0.1 ^bc^	0.5 ± 0.1 ^bc^	0.6 ± 0.1 ^ab^	0.6 ± 0.1 ^a^
Thr	3.6 ± 0.2 ^a^	3.6 ± 0.2 ^a^	3.4 ± 0.1 ^ab^	3.4 ± 0.1 ^ab^	3.3 ± 0.1 ^bc^	3.2 ± 0.1 ^c^
Val	0.5 ± 0.1 ^ab^	0.5 ± 0.1 ^ab^	0.5 ± 0.1 ^ab^	0.6 ± 0.1 ^a^	0.4 ± 0.1 ^b^	0.5 ± 0.1 ^ab^
Ala	1.0 ± 0.1 ^a^	1.0 ± 0.1 ^a^	0.8 ± 0.1 ^b^	0.8 ± 0.1 ^b^	0.8 ± 0.1 ^b^	0.7 ± 0.1 ^b^
Arg	3.1 ± 0.2 ^a^	3.2 ± 0.2 ^a^	2.3 ± 0.1 ^b^	2.3 ± 0.1 ^b^	2.4 ± 0.1 ^b^	1.9 ± 0.1 ^c^
Asp	3.0 ± 0.3 ^ab^	3.3 ± 0.3 ^a^	2.8 ± 0.1 ^b^	2.9 ± 0.1 ^ab^	3.3 ± 0.1 ^a^	3.1 ± 0 ^ab^
Glu	7.6 ± 0.2 ^a^	7.1 ± 0.1 ^b^	6.7 ± 0.3 ^c^	6.3 ± 0.3 ^d^	5.6 ± 0.1 ^e^	5.2 ± 0.2 ^f^
Gly	0.4 ± 0.1 ^a^	0.4 ± 0.1 ^a^	0.5 ± 0.1 ^a^	0.5 ± 0.1 ^a^	0.5 ± 0.1 ^a^	0.5 ± 0.1 ^a^
Pro	2.2 ± 0.2 ^ab^	2.2 ± 0.3 ^ab^	2.1 ± 0.1 ^ab^	2.0 ± 0.3 ^b^	2.0 ± 0.3 ^b^	1.9 ± 0.3 ^b^
Ser	1.4 ± 0.6 ^a^	1.5 ± 0.8 ^a^	1.3 ± 0.1 ^a^	1.6 ± 0.7 ^a^	1.4 ± 0.5 ^a^	1.5 ± 0.4 ^a^
Tyr	7.7 ± 0.1 ^a^	7.3 ± 0.5 ^a^	6.7 ± 0.1 ^b^	6.8 ± 0.3 ^b^	5.8 ± 0.9 ^c^	5.5 ± 0.8 ^c^
Cys-Cys	2.5 ± 0.2 ^c^	3.1 ± 0.2 ^b^	2.7 ± 0.1 ^bc^	2.9 ± 0.2 ^b^	3.8 ± 0 ^a^	3.7 ± 0 ^a^

CK: control group; HF: high-fat groups. Tryptophan was not determined (destroyed by acid hydrolysis). One-way analysis of variance (ANOVA) was used to conduct a comprehensive test for each column of indicators. Subsequently, the Tukey HSD test was used to conduct pairwise comparisons among all treatment groups (CK, HF-0, HF-0.01, HF-0.05, HF-0.1, and HF-0.2). In the same column, different superscript letters indicate significant differences between treatment groups (*p* < 0.05), while the same letter indicates no significant differences (*p* > 0.05).

**Table 4:** Fatty acid composition (% of total fatty acids) of BSFL under different dietary treatments: basal diet control (CK), high-fat control (HF-0), and high-fat diet supplemented with 0.01%, 0.05%, 0.1%, or 0.2% capsaicin (HF-0.01 to HF-0.2).

Composition	CK	HF-0	HF-0.01	HF-0.05	HF-0.1	HF-0.2
C10:0	0 ± 0 ^b^	0.5 ± 0.4 ^ab^	0 ± 0 ^b^	0.5 ± 0.5 ^ab^	1.3 ± 0.6 ^a^	1.3 ± 0.7 ^a^
C12:0	11.1 ± 1.6 ^c^	23.5 ± 2.0 ^b^	6.6 ± 1.0 ^d^	10.6 ± 1.8 ^c^	31.4 ± 1.8 ^a^	24.9 ± 1.7 ^b^
C13:0	0 ± 0	0 ± 0	0 ± 0	0 ± 0	0 ± 0	0 ± 0
C14:0	2.7 ± 1.0 ^bc^	4.6 ± 1.0 ^ab^	1.7 ± 0.6 ^c^	2.6 ± 0.6 ^bc^	5.5 ± 0.5 ^a^	4.5 ± 0.3 ^ab^
C14:1	0 ± 0	0 ± 0	0 ± 0	0 ± 0	0 ± 0	0 ± 0
C15:0	0.7 ± 0.7 ^a^	0.6 ± 0.6 ^a^	0.8 ± 0.6 ^a^	0.7 ± 0.6 ^a^	0.5 ± 0.4 ^a^	0.5 ± 0.4 ^a^
C16:0	18.3 ± 1.4 ^a^	17.5 ± 1.8 ^a^	19.2 ± 1.6 ^a^	19.8 ± 2.0 ^a^	17.6 ± 2.3 ^a^	18.8 ± 2.4 ^a^
C16:1	6.9 ± 1.3 ^ab^	5.6 ± 1.0 ^b^	9.3 ± 1.6 ^a^	9.0 ± 1.3 ^a^	6.6 ± 1.0 ^ab^	8.3 ± 1.2 ^a^
C17:0	0.9 ± 0.7 ^a^	0.6 ± 0.6 ^a^	1.3 ± 0.6 ^a^	1.1 ± 0.6 ^a^	0.7 ± 0.4 ^a^	0.9 ± 0.4 ^a^
C18:0	5.8 ± 1.0 ^a^	4.3 ± 1.0 ^bc^	6.4 ± 1.0 ^a^	5.1 ± 1.0 ^ab^	3.3 ± 1.0 ^c^	3.7 ± 1.0 ^c^
C18:1n-9	25.6 ± 1.0 ^a^	20.6 ± 1.3 ^b^	25.1 ± 1.4 ^a^	22.3 ± 1.8 ^b^	15.0 ± 2.0 ^c^	15.9 ± 2.2 ^c^
C18:2n-6	25.5 ± 1.8 ^a^	20.2 ± 1.8 ^c^	26.1 ± 2.0 ^a^	25.3 ± 1.4 ^ab^	15.4 ± 2.0 ^d^	18.0 ± 1.8 ^bc^
C18:3n-3	0.7 ± 0.7 ^a^	0.6 ± 0.6 ^a^	0.7 ± 0.6 ^a^	0.7 ± 0.4 ^a^	0.4 ± 0.4 ^a^	0.5 ± 0.3 ^a^
C18:3n-6	0 ± 0 ^a^	0 ± 0 ^a^	0 ± 0 a	0.1 ± 0.1 ^a^	0 ± 0 ^a^	0.1 ± 0.1 ^a^
C20:0	0.1 ± 0.1 ^a^	0 ± 0 ^a^	0.2 ± 0.2 ^a^	0.1 ± 0.1 ^a^	0 ± 0 ^a^	0.1 ± 0.1 ^a^
C20:4 n-6	0.3 ± 0.2 ^a^	0 ± 0 ^a^	0.6 ± 0.4 ^a^	0 ± 0 ^a^	0 ± 0 ^a^	0 ± 0 ^a^

CK: control group; HF: high-fat groups. All zero-value fatty acids (C13:0, C14:1) are retained to keep the table complete. One-way analysis of variance (ANOVA) was used to conduct a comprehensive test for each column of indicators. Subsequently, the Tukey HSD test was used to conduct pairwise comparisons among all treatment groups (CK, HF-0, HF-0.01, HF-0.05, HF-0.1, and HF-0.2). In the same line, different superscript letters indicate significant differences between treatment groups (*p* < 0.05), while the same letter indicates no significant differences (*p* > 0.05).

## Data Availability

The original contributions presented in this study are included in the article. Further inquiries can be directed to the corresponding author.
